# A Conference on Day Nursery Schools

**Published:** 1923-05

**Authors:** 


### A CONFERENCE ON DAY NURSERY SCHOOLS.

On the invitation of the Islington Borough Council a conference of over a hundred delegates from Metropolitan Borough Councils, including in each case the Medical Officer of Health, and various bodies devoted to children's welfare, met in the Islington Town Hall to consider the advisability of establishing Day Nursery Schools in the Metropolis on the lines of the Rachel McMillan School at Greenwich. The Mayor of Islington (Councillor S. C. Harper) was in the chair.

### Establishing a School.

Miss Margaret McMillan, C.B.E., said that in the establishment of any school there were three sources of expenditure to be considered—building and sites, staffing, and, in schools which provided food, diet. They wanted open spaces rather than buildings; therefore they had to look for sites. As regards staffing, it was found that one trained teacher to every 50 or 60 children was necessary, and for every group of six children a "pair of hands" and a "pair of eyes" are needed to assist in teaching and training the infants in good habits, &c.

### Cost of Feeding.

It was an illusion that diet cost very much. Practical experiment had shown that the full cost of feeding a child, giving him three meals a day, including a two-course dinner, amounted to half-a-crown a week. For that sum they could ensure that a child ran no risk of rickets or anæmia. She believed that the whole cost of a nursery school conducted on the best lines, giving every advantage, was not more than £14 or £15 per head. In well-to-do areas parents not only pay the cost of the feeding, but more if necessary.

### Beginning on a Large Scale.

In reply to various questions put during a lively discussion, Miss McMillan pointed out that it was essential to begin on a large scale, as the one stove, the one cook, and the one kitchen which would be required for small numbers would suffice to provide for over 200 children. To commence with only 20 or 30 children would be to court disaster. As regards the number in a shelter, she thought that 35 would be ideal, but they had 50. The L.C.C. infant school, where a sort of kindergarten education was given, was not a substitute for a Day Nursery School, which, in addition to educating, bathed and fed the child, and taught it to form good habits while it was under its care; it looked after the child from 8 a.m. to 5 p.m., during which time care was taken that it had a period of sleep, so essential at its age. The L.C.C. did not undertake such work, nor could they carry out a system of home visitation, to which the Day Nursery School attached so much importance.

### A Deputation to the L.C.C.

A motion put by Alderman Varley, to the effect that the conference recommends that the principle of Day Nursery Schools should be extended to other parts of London was carried unanimously. It was also resolved to ask the London County Council to receive a deputation from the conference to discuss the scheme.

